# A Unique Case of Drug Interaction between Ticagrelor and Statin Leading to Acute Renal Failure

**DOI:** 10.7759/cureus.1633

**Published:** 2017-08-31

**Authors:** Gbeminiyi Samuel, Adebayo C Atanda, Alex Onyemeh, Ahmad Awan, Oyintayo Ajiboye

**Affiliations:** 1 Department of Internal Medicine, Howard University Hospital; 2 Public Health, John Hopkins School of Public Health

**Keywords:** rhabdomyolysis-induced renal failure, ticagrelor, high intensity statin, post myocardial infarction

## Abstract

Dual antiplatelet agents and high-intensity statins are frequently used in combination after myocardial infarction. Ticagrelor has the potential of causing acute kidney injury. Rosuvastatin is excreted through the kidneys and dose adjustment is needed in patients with kidney disease. When used in combination, they can potentiate the toxic effects of each other. We report a case of drug interaction between rosuvastatin and ticagrelor resulting in rhabdomyolysis and acute renal failure necessitating dialysis. This case stresses the importance of monitoring renal function and adjusting the dose of rosuvastatin accordingly in patients with kidney disease.

## Introduction

Ticagrelor is a potent antiplatelet drug that, when compared with clopidogrel, was found to significantly reduce the rate of death from vascular causes, myocardial infarction, or stroke without an increase in the rate of overall major bleeding in the Platelet Inhibition and Patient Outcomes (PLATO) trial. Ticagrelor has shown to cause acute kidney injury, which can lead to elevated levels of rosuvastatin, which is excreted through the kidneys. We present a case of rhabdomyolysis and acute kidney failure caused by the combination of ticagrelor and rosuvastatin. This case highlights the importance of closely monitoring renal functions in patients who are on ticagrelor and adjusting the dose of rosuvastatin accordingly in patients with kidney disease.

## Case presentation

A 49-year-old African American female with a past medical history of hypertension, diabetes mellitus, discoid lupus, remote history of post-streptococcal glomerulonephritis with complete recovery, and multivessel disease with drug-eluting stent placement presented with generalized weakness of three days duration. Of note, the patient reported compliance with a three day per week rigorous exercise routine at a cardiac rehabilitation center resulting in a 20 lb weight loss in a couple of months after percutaneous coronary intervention. She was transferred to our facility for further care from a neighboring hospital after she presented with a three-day history of diarrhea, abdominal cramps, chest pain, and anuria.

The patient was on lisinopril 20 mg oral daily, metformin 500 mg oral daily, metoprolol tartrate 50 mg oral two times daily, pantoprazole 40 mg oral daily, rosuvastatin 20 mg oral daily, and ticagrelor 90 mg oral daily.

On physical examination, the patient appeared toxic. A cardiovascular examination was normal except for reproducible left chest tenderness. There was an area of ecchymosis and erythema about 6 cm in its widest diameter on the lateral part of the right thigh, where she landed on after a fall due to progressive weakness. The laboratory values at the time of arrival was significant for sodium of 126 mEq/l, blood urea nitrogen of 58 mg/dl, creatinine of 9.1 mg/dl with a baseline creatinine from last admission of 1.0 mg/dl, creatine phosphokinase (CPK) of 238,678 IU/l, myoglobin of 46,208 ng/ml, and troponin of 1.36 ng/ml. The brain natriuretic peptide count was 10,518 pg/ml. The white cell count was 13,500 cells/mm3. The lactic acid level was 2.2 mm/l, and the significant liver function test done the previous day from the previous hospital admission per their records showed an aspartate aminotransferase count of 2000. The patient was admitted to the medical intensive care unit (MICU) and was managed for rhabdomyolysis. The poor urine output limited the use of intravenous fluid for the management of rhabdomyolysis. Nephrology was consulted for emergent dialysis to manage anuria and rhabdomyolysis.

**Figure 1:**
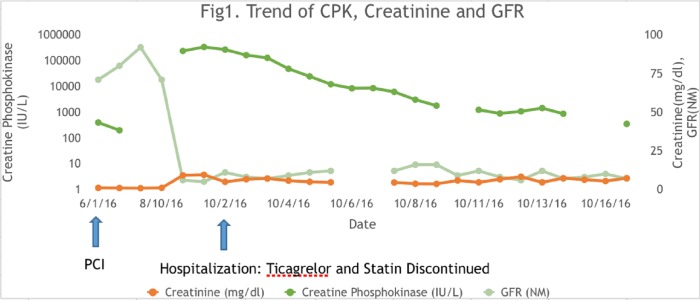
Trend of creatinine phosphokinase and creatinine after percutaneous coronary intervention CPK: creatinine phophokinase, PCI: percutaneous coronary intervention.

Peak CPK level was attained on Day 2 of intensive care unit admission at 338,601 IU/ml. The trend of creatinine, glomerular filtration rate, and CPK since the last percutaneous intervention is shown in Figure 1.

Rosuvastatin and ticagrelor were immediately stopped as ticagrelor increased the rosuvastatin to toxic levels in the body, causing increased hepatic enzyme levels and rhabdomyolysis. The patient was then placed on clopidogrel. Over the next four days, the patient's improvement was significant. The patient had received four hemodialysis sessions and her CPK levels were trending down exponentially. Her urine output improved. She was discharged home on aspirin and clopidogrel with the recommendations to re-evaluate for starting rosuvastatin on outpatient basis after the resolution of acute kidney injury.

## Discussion

Ticagrelor blocks the binding of adenosine diphosphate to a specific platelet receptor P2Y12, thereby inhibiting platelet activation [1]. Ticagreolor is a potent antiplatelet drug, and in the Platelet Inhibition and Patient Outcomes (PLATO) trial, when compared with Plavix, was found to significantly reduce the rate of death from vascular causes, myocardial infarction, or stroke without an increase in the rate of overall major bleeding [2]. Ticagrelor is metablolized through cytochrome 450 (CYP 450) 3A4. Fifty-eight percent of ticagrelor is excreted through the gastrointestinal tract and 27% via urine. Both the first drug and active metabolite have antiplatelet properties allowing for rapid and effective antiplatelet activity [3].

In the PLATO trial, serum creatinine concentration increased by more than 30% in more than 25.5% of the patients receiving ticagrelor. The exact mechanism leading to worsening renal function is unknown. However, it was found to be more pronounced in patients over 75 years of age, in patients receiving angiotensin receptor inhibitors, and patients with pre-existing renal dysfunction [2-4].

Ticagrelor and high intensity statins are routinely prescribed in patients with myocardial infarction. Both are metabolized through the cytochrome system. Rhabdomyolysis is a rare but clinically important side effect of statin use, either alone or in combination with drugs that increase the potency of statins [5]. The metabolism of rosuvastatin appears to be less significant by 3A4 system and it is mostly through the 2C9 enzyme [6]; because of this, it has less significant drug-drug interactions. The dosage of rosuvastatin should be adjusted in renal failure since its plasma concentration may increase threefold with an estimated glomerular filtration rate less than 30 ml/min [3].

Ticagrelor by itself doesn’t cause rhabdomyolysis, although it does cause worsening of renal function by unspecified mechanism. It is therefore reasonable to presume that in our patient the worsening renal function led to an increased level of rosuvastatin, which led to rhabdomyolysis and worsening of the kidney function, necessitating dialysis in the acute setting.

Although, ticagrelor can cause worsening of the kidney function, no dose adjustment is required in a patient with renal dysfunction [7]. However, the dosage of rosuvastatin should be decreased in patients with less than normal glomerular filtration rate. This case report highlights the importance of checking renal functions regularly and adjusting the rosuvastatin dosage accordingly.

## Conclusions

Ticagrelor and rosuvastatin are frequently used in combination for the secondary prevention of coronary artery disease especially after an acute coronary syndrome. Clinicians should maintain a high index of suspicion for drug interaction causing rhabodmyolysis, which may be precipitated as well as aggravated by rigorous exercise and dehydration.
